# Variable use of amiodarone is associated with a greater risk of recurrence of atrial fibrillation in the critically ill

**DOI:** 10.1186/s13054-016-1252-2

**Published:** 2016-04-02

**Authors:** Goran Mitrić, Andrew Udy, Hiran Bandeshe, Pierre Clement, Rob Boots

**Affiliations:** School of Medicine, University of Queensland, Brisbane, QLD Australia; Department of Intensive Care Medicine, Royal Brisbane & Women’s Hospital, Herston, Brisbane, QLD Australia; Burns Trauma and Critical Care Research Centre, University of Queensland, Brisbane, QLD Australia; Department of Intensive Care and Hyperbaric Medicine, The Alfred Hospital, Prahran, VIC Australia; Department of Epidemiology and Preventive Medicine, Monash University, The Alfred Centre, Prahran, Melbourne, VIC Australia

**Keywords:** Amiodarone, Atrial fibrillation, Critical care, Recurrence

## Abstract

**Background:**

Atrial fibrillation is a common rhythm disturbance in the general medical-surgical intensive care unit. Amiodarone is a popular drug in this setting but evidence to inform clinical practice remains scarce. We aimed to identify whether variation in the clinical use of amiodarone was associated with recurrent atrial fibrillation.

**Methods:**

This was a retrospective audit of 177 critically ill patients who developed new-onset atrial fibrillation after admission to a tertiary level medical-surgical trauma intensive care unit. Patterns of amiodarone prescription (including dosage schedule and duration) were assessed in relation to recurrence of atrial fibrillation during the intensive care unit stay. Known recurrence risk factors, such as inotrope administration, cardiac disease indices, Charlson Comorbidity Index, magnesium concentrations, fluid balance, and potassium concentrations, were also included in adjusted analysis using forward stepwise logistic regression modelling.

**Results:**

The cohort had a median (interquartile range) age of 69 years (60–75), Acute Physiology and Chronic Health Evalution II score of 22 (17–28) and Charlson Comorbidity Index of 2 (1–4). A bolus dose of amiodarone followed by infusion (*P* = 0.02), in addition to continuing amiodarone infusion through to discharge from the intensive care unit (*P* < 0.001), were associated with less recurrent dysrhythmia. Recurrence after successful treatment was associated with ceasing amiodarone while an inotrope infusion continued (*P* < 0.001), and was more common in patients with a prior history of congestive cardiac failure (*P* = 0.04), and a diagnosis of systemic inflammatory response syndrome (*P* = 0.02).

**Conclusions:**

Amiodarone should be administered as a bolus dose followed immediately with an infusion when treating atrial fibrillation in the medical-surgical intensive care unit. Consideration should be given to continuing amiodarone infusions in patients on inotropes until they are ceased.

## Background

Atrial fibrillation (AF) is a common cardiac rhythm disturbance encountered in critically ill patients in the general medical or surgical intensive care unit (ICU) [1]. Depending upon the study population, AF is reported to occur at frequencies ranging from 8.3 % to 46 %, the latter being associated with patients who have undergone cardiac surgery or were admitted for treatment of sepsis [1–9]. The occurrence of AF potentially leads to thromboembolism or haemodynamic compromise [10]. Risk factors for the development of AF in critically ill patients have been predominantly derived from cardiothoracic surgical patients. Data on risk factors for atrial fibrillation in the non-cardiothoracic surgery ICU population are scarce.

Defined risks include: use of catecholamines and positive inotropic drugs [8, 11, 12], high severity of disease index scores [1, 5, 9, 13], sepsis [1, 5], cardiovascular disease [4, 9, 12, 13], electrolyte disturbances [4], advanced age [9, 12, 13], elevated markers of inflammation [5, 6], hypoxia [5], and high central venous pressures [5]. Whether part of the patient’s prior history, new in onset or recurrent, AF has been shown to be an independent risk factor for mortality in the ICU [3, 13–15]. Of note, new-onset AF has been associated with increased diastolic dysfunction, vasopressor use, and a greater cumulative positive fluid balance [15], although similar data are not widely available for recurrent AF in the ICU.

Amiodarone is considered the drug of choice for the treatment of AF in the ICU [16]. Two randomised controlled trials have shown amiodarone to be effective in converting AF into sinus rhythm in this setting [17, 18]. The efficacy of amiodarone has also been confirmed by other studies showing that it is effective at both converting AF into sinus rhythm [17–20] and controlling ventricular rate [21, 22], while being haemodynamically well tolerated [23, 24]. Many dosing regimens have been described with no consensus on the optimal treatment strategy. Therefore, the present study aimed to assess the effect of variation in amiodarone use on dysrhythmia recurrence in patients with new-onset AF admitted to a non-cardiothoracic ICU.

## Methods

We conducted a retrospective cohort study in a large general medical-surgical ICU over a 24-month period. Data were recorded prospectively in electronic format (IntelliVue Clinical Information Portfolio, Philips Medical Systems, Eindhoven, Netherlands). Patients were eligible for inclusion if they were adults (18 years or older) who developed new-onset AF during their ICU stay and were treated with amiodarone. Exclusion criteria were recent cardiothoracic surgery, inadequate records being available for analysis, previous treatment with amiodarone at any time point prior to entry into the study, and a previous history of atrial fibrillation.

### Definitions and data collection

AF was defined as a rhythm on the electrocardiogram (ECG) with replacement of P waves with rapid oscillations or fibrillatory waves that vary in size, shape and timing, associated with an irregular, frequently rapid, ventricular response when atrioventricular conduction is intact [25]. This was derived from the confirmed hourly recordings of cardiac rhythm from the clinical information system as reported from the algorithm analysis programme of the Phillips IntelliVue IP critical care monitoring system. Successful treatment was defined as conversion of AF into normal sinus rhythm within 12 hours of amiodarone administration. Recurrence was defined as AF identified on the ECG occurring prior to discharge from the ICU following successful conversion of cardiac rhythm from AF into normal sinus rhythm. At the time of AF, the presence of hypomagnesaemia (Mg <0.8 mmol/L), hypokalaemia (K <3.5 mmol/L), the presence of a systemic inflammatory response syndrome (SIRS) [26], and sepsis (infection as a presumed or proven cause of SIRS) [27], were recorded.

In summarising the use of amiodarone, a bolus was defined as a fixed dose of greater than 150 mg given over 20 minutes to an hour, a continuous infusion was a fixed dose of amiodarone delivered hourly by a syringe pump for more than 2 hours, and delay to an infusion was a gap of 1 hour in the fluid administration record for the administration of a bolus and the commencement of an infusion.

Continuous automated ECG rhythm tracings were recorded for all patients for the entire duration of their ICU stay. Data collection included records of drugs administered (amiodarone and all inotropes), their doses, and infusion rates. Physiologic variables were recorded daily including fluid balance, white cell count, central venous pressure, and temperature. Demographic data included age, gender, weight, height, body mass index (BMI) [28], Acute Physiology and Chronic Health Evaluation II (APACHE II) score [29], Simplified Acute Physiology Score II (SAPS II) [30], ICU admission and discharge times, hospital admission and discharge times, hospital outcome, and ICU outcome. The time and date for AF onset, conversion to normal sinus rhythm, and any recurrence were extracted from ECG data. Data regarding comorbidities were collected using the Charlson Comorbidity Index [31] with both scores and diagnoses recorded. The presence of past rheumatic fever [32], uncontrolled hyperthyroidism, mitral valve disease, haemochromatosis [33], hypertension [34] and concurrent use of digoxin and metoprolol were also recorded. If patients had multiple admissions during the study period only the initial admission in which amiodarone was used for the first time was included in data collection.

The study was granted low-risk research approval by the Human Research Ethics Committee, Royal Brisbane and Women's Hospital as well as the Medical Research Ethics Committee, University of Queensland with individual consent waived due to the retrospective study design (HREC/11/QRBW/292 and 2012000135).

### Analysis plan

Descriptive statistics were calculated for all study variables, with data reported as means or medians with interquartile ranges (IQRs) for continuous data, percentages for categorical data, and 95 % confidence intervals where appropriate. Data were analysed using Kruskal-Wallis and Wilcoxon sign rank tests for continuous data where appropriate. Categorical data were assessed using chi squared or Fisher’s exact test where analysis assumptions were met. No assumptions were made for missing data and proportions were adjusted for the number of patients with available data. A two-sided *P* value of less than 0.05 was considered to indicate statistical significance. Forward stepwise logistic regression modelling for variables predictive of recurrence of AF was used. Variables were included if they were recognised as prognostic for recurrence of AF in critically ill patients, or if their *P* value was < 0.2 in univariate testing. Discrete variables were included as bipolar outcomes and continuous variables approximated a normal distribution or were collapsed into an ordinal variable. Models were assessed for discrimination using the area under the receiver-operating function and goodness of fit (Hosmer-Lemeshow). Data were analysed using Stata 9 Statistical package (College Station, TX, USA).

## Results

**Fig. 1:**
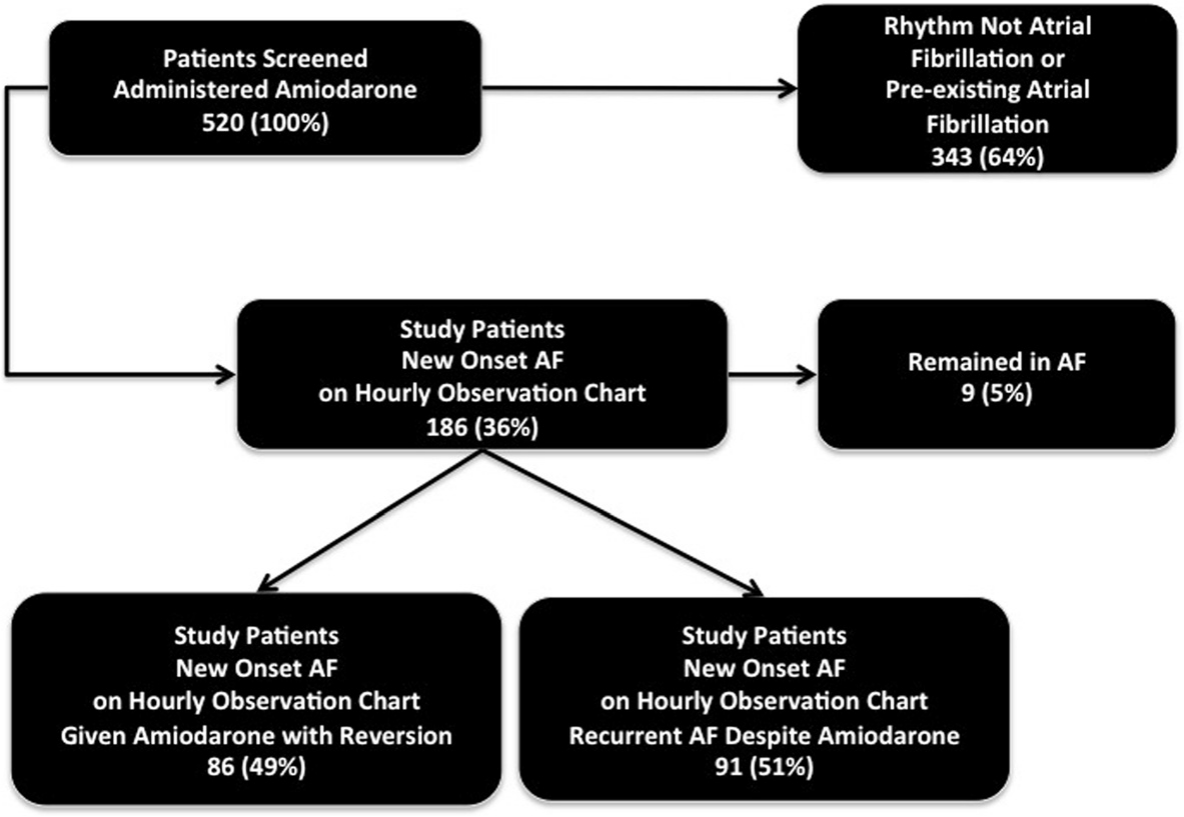
Consort Diagram for Atrial Fribrillation Patients Admitted to ICU. *AF* Atrial fibrillation

Over the 2-year study period, we identified 520 admissions in which patients were administered amiodarone for AF at some point during their ICU stay. Of these, 186 met criteria for study inclusion. Of these, 86 (49 %) were successfully treated with amiodarone, without recurrence of AF until discharge from ICU. Nine patients remained in AF until discharge from the ICU and were not considered further in the analysis. In the remaining 91 patients (51 %), there was recurrence of AF at least once during the ICU stay, after initial successful conversion to normal sinus rhythm (Fig. 1).

The median (IQR) age of patients was 69 years (60–75), they were predominantly male (64 %), with a median APACHE II score of 22 (17–28). Baseline characteristics of patients including demographic information, severity of disease indices and co-morbidities are presented in Tables 1 and 2. Echocardiogram information was only available in 33 patients, too small a number to include in any detailed analysis. All four patients with hyperthyroidism had recurrent AF. Patients with or without recurrent AF concurrently received digoxin (27 % compared to 10 %, *P* = 0.004) or beta-blockers (46 % compared to 53 %, *P* = 0.37) with all patients receiving digoxin also receiving beta-blockers, reflecting the use of multiple agents to control recurrent AF. The majority of patients received noradrenaline as an inotrope (three without AF recurrence and five with AF recurrence received adrenaline). No patients were electrically cardioverted or received dopamine or dobutamine.

**Table 1:** Patient characteristics

Parameter	No recurrence of AF (*n* = 86)	Recurrence of AF (*n* = 91)	Total (*n* = 177)	*P*
Age (years)	65 (57–75)	71 (61–76)	69 (60–75)	0.10
Male	53 (61)	60 (66)	113 (64)	0.64
APACHE II	21 (17–26)	23 (17–29)	22 (17–28)	0.28
SAPS II	39 (30–49)	44 (31–58)	41 (31–53)	0.12
ICU LOS (days)	6 (3–12)	8 (4–16)	7 (4–13)	0.05
Hospital LOS (days)	21 (12–46)	31 (18–70)	25 (13–58)	0.09
ICU Outcome				0.83
Died	10 (12)	13 (14)	23 (13)	
Discharged	75 (87)	77 (85)	152 (86)	
Transferred	1 (1)	1 (1)	2 (1)	
Hospital Outcome				0.08
Died	22 (26)	25 (27)	47 (27)	
Discharged Home	44 (51)	37 (41)	81 (46)	
Transferred	17 (20)	29 (32)	46 (26)	
Not recorded	3 (3)	0 (0)	3 (2)	
Weight (kg)	80 (69–100) (*n* = 79)	75 (65–90) (*n* = 87)	78 (66–95) (*n* = 166)	0.15
Height (cm)	170 (165–175) (*n* = 76)	170 (162–175) (*n* = 84)	170 (162–175) (*n* = 160)	0.73
Body Mass Index	28 (25–34) (*n* = 76)	26 (24–31) (*n* = 84)	28 (24–33) (*n* = 160)	0.06
Charlson Score	2 (1–4)	3 (2–5)	2 (1–4)	0.01
Concurrent use of digoxin*	9 (10)	25 (27)	34 (19)	0.004
Concurrent use of beta-blockers	46 (53)	42 (46)	88 (50)	0.37

* All patients receiving digoxin were also on a beta-blocker

Values are shown as median (IQR) or number (%) as appropriate

*AF* Atrial fibrillation, *APACHE* Acute Physiology and Chronic Health Evaluation, *BMI* body mass index, *ICU* intensive care unit, *LOS* length of stay, *SAPS* Simplified Acute Physiology Score

**Table 2:** Comorbidities

Comorbidity	No recurrent AF (*n* = 86)	Recurrent AF (*n* = 91)	Total (*n* = 177)	*P*
Myocardial infarction	17 (20)	26 (29)	43 (24)	0.22
Congestive cardiac failure	6 (7)	16 (18)	22 (12)	0.04
Peripheral vascular disease	14 (16)	26 (29)	40 (23)	0.07
Cerebrovascular disease	13 (15)	6 (7)	19 (11)	0.09
Dementia	4 (5)	1 (1)	5 (3)	0.20
Chronic pulmonary disease	26 (30)	32 (35)	58 (33)	0.52
Connective tissue disease	4 (5)	7 (8)	11 (6)	0.54
Peptic ulcer disease	5 (6)	4 (4)	9 (5)	0.74
Mild liver disease	3 (3)	6 (7)	9 (5)	0.74
Diabetes mellitus	14 (16)	11 (12)	25 (14)	0.52
Moderately severe renal disease	14 (16)	16 (18)	30 (17)	0.84
Diabetes mellitus – severe	3 (3)	3(3)	6 (3)	1.00
Any tumour	11 (13)	14 (15)	25 (14)	0.67
Leukaemia	3 (3)	9 (10)	12 (7)	0.13
Lymphoma	2 (2)	2 (2)	4 (2)	1.00
Moderately severe liver disease	1 (1)	2 (2)	3 (2)	1.00
Metastatic tumour	3 (3)	9 (10)	12 (7)	0.13
SIRS	47 (55)	62 (68)	109 (62)	0.09
Sepsis	28 (33)	36 (40)	64 (36)	0.35
Ischaemic heart disease	23 (27)	35 (38)	58 (33)	0.11
Hypertension	50 (58)	56 (62)	106 (60)	0.65
Hypokalaemia	29 (34)	31 (34)	60 (34)	1.00
Rheumatic heart disease	2 (2)	0 (0)	2 (1)	0.24
Mitral valve disease	7 (8)	2 (2)	9 (5)	0.09
Haemochromatosis	1 (1)	1 (1)	2 (1)	1.00

Values are shown as number (%)

*AF* Atrial fibrillation, *SIRS* systemic inflammatory response syndrome

Patients with recurrent AF had a higher Charlson Comorbidity Index and were more likely to have a history of cardiac failure (Table 2). Age and severity of illness indices were not significantly associated with recurrence of AF. It was not possible to define a suitable time frame for the comparison of physiological AF risk factors in the group that did not have a recurrence. As such, a within-group analysis of physiological parameters was undertaken in those with recurrent AF, comparing variables at the time of AF recurrence and the time of initial reversion to normal sinus rhythm. For patients with recurrent AF, a less positive 24-hour fluid balance, lower serum magnesium concentrations and a higher white cell count were observed on the day of AF recurrence (Table 3).

**Table 3:** Summary of physiological parameters in patients with recurrent atrial fibrillation

Parameter	On day atrial fibrillation initially reverted	On day atrial fibrillation recurred	*P*
24-hour fluid balance (ml)	963 (223–2010) (*n* = 82)	173 (−847 to 1304) (*n* = 76)	0.001
CVP min (cmH_2_O)	7 (4–10) (*n* = 57)	6 (3–9) (*n* = 64)	0.75
CVP max (cmH_2_O)	19 (14–22) (*n* = 57)	18 (13–22) (*n* = 64)	0.10
K min (mmol/L)	4.2 (3.8–4.5) (*n* = 81)	4.2 (4.0–4.6) (*n* = 73)	0.18
K max (mmol/L)	4.3 (4.1–4.7) (*n* = 81)	4.4 (4.1–4.7) (*n* = 73)	0.86
Mg min (mmol/L)	1.07 (0.91–1.26) (*n* = 81)	1.03 (0.92–1.18) (*n* = 71)	0.23
Mg max (mmol/L)	1.15 (0.99–1.34) (*n* = 81)	1.08 (0.96–1.24) (*n* = 71)	0.03
Temperature min (°C)	36.5 (36–37.1) (*n* = 82)	36.4 (36–36.9) (*n* = 76)	0.34
Temperature max (°C)	37.8 (37.1–38.4) (*n* = 82)	37.6 (37–38.4) (*n* = 76)	0.36
WCC min (×10^9^/L)	10.7 (7.5–15.6) (*n* = 78)	12.2 (9.8–18.2) (*n* = 71)	0.01
WCC max (×10^9^/L)	11.3 (7.8–15.9) (*n* = 78)	12.5 (9.8–18.7) (*n* = 71)	0.27

Values are shown as median (IQR), with missing data for differing patient numbers

*CVP* Central venous pressure, *K* potassium concentration, *max* maximum, *Mg* magnesium concentration, *min* minimum, *WCC* white cell count

There was no uniform treatment strategy with the use of amiodarone (Table 4). In 62 (35 %) patients, no bolus dose or prior administration of amiodarone was recorded. Only 92 (52 %) patients received both bolus dosing and an infusion. The median (IQR) total dose of amiodarone delivered was 905 mg (488–1651) which included both bolus doses and infusions, with a median duration of treatment of 24 hours (16–40 hours). The median delay to infusion after bolus was 2 hours (1–4). Patients receiving a bolus of amiodarone or an infusion only were more likely to have a recurrence of AF (*P* < 0.001). Patients with recurrence received more amiodarone overall, which may be due to persisting risk factors requiring longer treatment or greater amiodarone dosing.

**Table 4:** Amiodarone therapy summary

Parameter	No AF recurrence (*n* = 86)	AF recurrence (*n* = 91)	Total (*n* = 177)	*P*
Amiodarone boluses				0.07
0	43 (42)	19 (25)	62 (35)	
1	51 (50)	47 (61)	98 (55)	
2	5 (5)	7 (9)	12 (7)	
3	2 (2)	3 (4)	5 (3)	
Amiodarone dosing				<0.001
Bolus only	3 (3)	20 (23)	23 (13)	
Infusion only	43 (43)	19 (25)	62 (35)	
Bolus and infusion	40 (47)	52 (57)	92 (52)	
Delay to infusion after bolus (hours)	2 (1–3) (*n* = 29)	2 (1–6) (*n* = 45)	2 (1–4) (*n* = 74)	0.48
Total dose amiodarone (mg)	702 (300–1117)	1366 (752–2711)	905 (488–1651)	<0.001
Infusion time (hours)	20 (12–28)	31 (20–58)	24 (16–40)	<0.001
Continuing to receive inotropes with amiodarone ceased	0 (0) (*n* = 2)	23 (66) (*n* = 35)	37 (100) (*n* = 37)	0.14

Values are shown as median (IQR) or number (%) as appropriate

*AF* Atrial fibrillation

In logistic regression modelling, patients who were receiving inotropes during treatment for AF were found to have an increased risk of recurrence when amiodarone was stopped prior to cessation of inotrope infusion (*P* < 0.001; Table 5). Receiving amiodarone for the entire duration of the ICU stay once AF developed was associated with a lower risk of recurrence of AF (*P* < 0.001), as was receiving an initial bolus of amiodarone followed by an infusion over 24 hours, rather than just an infusion without a bolus dose (*P* = 0.02). If patients had a prior history of congestive cardiac failure, they were more likely to have a recurrence (*P* = 0.04). The diagnosis of SIRS was also a significant predictor of AF recurrence (*P* = 0.02). Although the use of digoxin was associated with recurrence, this was not found to be an independent predictor and more in keeping with the use of an additional agent to control the recurrence.

**Table 5:** Logistic regression of risk factors for recurrence of atrial fibrillation

Variable	OR	95 % CI	*P*
Univariate regression			
History congestive cardiac failure	2.84	1.05–7.65	0.04
Peripheral vascular disease	2.06	0.99–4.27	0.05
Cerebrovascular disease	0.40	0.14–1.10	0.07
SIRS	1.77	0.96–3.27	0.07
Ischaemic heart disease	1.71	0.91–3.23	0.10
Mitral valve disease	0.25	0.05–1.26	0.09
Metastatic tumour	3.03	0.79–11.6	0.11
Leukaemia	3.03	0.79-11.61	0.11
Age	1.02	1.00–1.04	0.12
Body mass index	0.96	0.92–1.00	0.06
Concurrent use of beta-blockers	1.50	0.83-2.73	0.18
Concurrent use of digoxin*	5.06	2.20–11.67	<0.001
Ceasing amiodarone while on inotrope infusion	8.76	3.58–21.4	<0.001
Delay of infusion after bolus dose of amiodarone	1.86	1.02–3.44	0.05
Receiving a bolus dose and infusion of amiodarone	0.44	0.23–0.86	0.02
Remaining on amiodarone to discharge from ICU	0.02	0.01–0.05	<0.001
Multivariate regression			
Ceasing amiodarone while on inotrope infusion	5.89	1.86–18.6	0.003
Remaining on amiodarone for duration of ICU admission	0.01	0.003–0.04	<0.001
SIRS	4.21	1.32–13.4	0.02
Hosmer and Lemeshow Goodness of Fit Chi^2^ 7.19, *P* = 0.21			
Area under ROC curve (discrimination) 0.92			

* All patients receiving digoxin also were on a beta-blocker

*CI* Confidence interval, *ICU* intensive care unit, *OR* odds ratio, *ROC* receiver operating characteristic, *SIRS* systemic inflammatory response syndrome

## Discussion

We identified several clinically significant factors associated with recurrence of new-onset AF in our cohort of patients. Ceasing amiodarone in patients who were still receiving inotrope infusions was associated with AF recurrence. Patients who remained on amiodarone treatment for the entire duration of their ICU stay once AF developed were less likely to have recurrence of AF. Similarly, AF recurrence was less likely in patients in whom a bolus dose and infusion of amiodarone was used rather than an infusion without an initial bolus. The risk of recurrence was also significantly associated with a prior history of congestive cardiac failure and the presence of SIRS.

The electrophysiological changes that occur in the atrium in AF have been extensively studied but remain poorly understood, especially in the critically ill. It is thought that AF is initiated through focal (ectopic) activity in the left atrium near the pulmonary veins. The mechanism underlying this is believed to be premature firing of action potentials through acceleration of phase four depolarisation (abnormal automaticity) as well as early and delayed after-depolarisations (EAD and DAD, respectively) [35]. Both of these mechanisms have been shown to be initiated through adrenergic stimulation with catecholamines [35]. It has also been shown that AF is maintained through a process termed multiple wavelet intramural re-entry, in which there is a continuous re-entry circuit formed within the atria [35, 36]. This effect has been shown to be stimulated by adrenergic drugs [35, 36, 37]. In keeping with these findings, we have shown that patients on inotrope infusions had higher rates of recurrence when amiodarone was ceased while these agents continued.

Amiodarone is considered to be the drug of choice for treating AF [16], although there is little evidence of its utility in a general ICU population. Despite having beta adrenergic blocking activity, it is unlikely to affect catecholamine dose requirements [23]. To our knowledge, only two randomised controlled trials have specifically assessed the use of amiodarone for treatment of AF in general ICU patients. Chapman et al. [17] evaluated the efficacy of amiodarone for AF in non-cardiothoracic critically ill patients in a randomised controlled trial compared with procainamide. The dose of amiodarone in this study was based on weight (3 mg/kg bolus followed by 10 mg/kg/24 hours) and the rate of conversion into sinus rhythm was 70 % in the amiodarone group. Drugs were given for a minimum of 72 hours. We found that patients who developed AF and were treated with amiodarone for the entire duration of their ICU admission were less likely to have a recurrence of AF, as at this time inciting factors presumably had resolved. Our study supports the use of an adequate loading dose followed by an infusion of amiodarone, with continuation until inotropes are ceased, in order to reduce the risk of recurrence of AF.

A longer duration of treatment with amiodarone may result in a greater likelihood of rhythm stability. Moran et al. [18] performed a randomised controlled trial assessing the efficacy of amiodarone compared with magnesium sulphate. This study found that amiodarone was successful at converting AF into sinus rhythm in 7/14 (50 %) patients. Interestingly, this study found amiodarone to be less effective than Chapman et al. [17], despite higher doses of amiodarone being used (5 mg/kg bolus followed by 10 mg/kg/24 hours). Both these studies included tachyarrhythmias other than AF, and may account for the differences in rates of reversion to sinus rhythm. Both of these trials lacked a follow-up duration suitable to identify patients with recurrences, nor explored recurrence risk factors.

A wide range of dosing regimens are used for treating AF (3–7.5 mg/kg bolus and follow on infusions of 1200–1500 mg/24 hours or 10 mg/kg/24 hours) [17–20, 23]. This lack of consensus on the best dosing regimen was reflected in our results. There was no uniform dosing strategy used and 35 % of patients did not receive a loading dose. Patients receiving an amiodarone bolus typically received an “ampoule” of 300 mg regardless of weight and had a median delay in a follow-on infusion of 2 hours. Patients receiving a loading dose and an infusion were less likely to have recurrence when compared to those in whom only a continuous infusion without a bolus was administered.

Several studies have reported risk factors for development of AF in the critically ill patient, mainly in the cardiothoracic surgical setting. Cardiovascular disease has previously been implicated in the development of AF. Factors which are predictive of increasing risk include coronary artery disease [4], cardiomegaly [4], low left ventricular ejection fraction [9, 12], pre-existing cardiovascular disease [13], and right ventricular dysfunction [12]. Other studies have suggested inflammatory processes as a risk factor for AF, with SIRS [5], sepsis [5] and a raised C reactive protein [6] being implicated. Consistent with these, we found AF recurrence associated with congestive cardiac failure, altered fluid balance, low serum magnesium and an elevated white cell count, the latter supportive of the association with SIRS. The two patients with hyperthyroidism were not suspected clinically of such at the time of amiodarone administration.

Physiologic disturbances, such as electrolyte derangements, commonly precipitate rhythm disorders such as AF [4]. We assessed maximum and minimum values, as the timing of the measures in this retrospective analysis were quite variable, potentially masking an association if daily averaged or time averaging values were used. Abnormal values are unlikely to persist for long without correction in an intensive care environment. Our study examined patients once they developed AF, and hence electrolytes and other physiologic variables would presumably be kept strictly within normal limits. Positive fluid balance potentially causing atrial stretch is well recognised as a risk factor for AF. As for patients with recurrence, deciding on the relevant cumulative fluid balance to compare patients with and without recurrence at a similar point of time in their disease is difficult. In our study, a less positive 24-hour fluid balance at the time of AF recurrence was noted. Dynamic changes in the degree of atrial stretch may be just as important as any specific degree of stretch at a single point in time as a cause for AF. This would be better assessed prospectively with more objective assessment of cardiovascular fill such as inferior vena cava dimensions and changes with respiration on ultrasound imaging, as well as the effect of the time frame of changes in volume status.

### Strengths and limitations

Our study was performed in a tertiary referral hospital with a wide range of specialist surgical and medical services excluding cardiac surgery. Specialist services included bone marrow transplantation, obstetric, trauma and burns units. Our findings are unique as no previous studies have specifically examined factors leading to AF recurrence once successfully treated.

Our study can only make inference about patients receiving amiodarone, as patients who received other pharmacological treatments and electrical cardioversion without amiodarone were excluded. The effects of concomitant use of beta-blockers or digoxin were not significant in multivariate modelling. Other treatment options such as intravenous diltiazem are not available in Australia [38].

Other than in the setting of paroxysmal AF [39], there is no agreed time period for which the patient must remain in sinus rhythm following cardioversion from AF in order to define this as successful. In this study, we pragmatically used 12 hours of sinus rhythm as the minimal time for successful treatment with amiodarone. This reflects the acuteness of illness within an ICU and is an average of studies of AF in the critically ill where the efficacy of therapy has been assessed within 2 hours and 24 hours of drug administration [40].

Our study had a relatively small sample size as our focus was on the use of amiodarone. As such, only known risk factors were included in an attempt not to overfit the regression model. Ventilation parameters such as level of positive end-expiratory pressure were not considered as they often reflect fluid status and are unlikely to interact with recurrence. Early death may have been influenced by chance to detect AF recurrence; however, ICU duration of stay and the ICU mortality were similar with complete follow-up for the period of the ICU admission in both groups. Despite this, we were able to show statistically significant associations with recurrence of AF. Our project was also retrospective in nature, limiting the choice of variables to record for analysis. However, relevant clinical data were available and recorded prospectively in a systematic manner in an electronic clinical information system.

## Conclusion

Patients with new-onset AF who are treated with amiodarone should receive a loading dose, immediately followed by an infusion. Clinicians should consider continuing amiodarone infusions in patients who are receiving inotropes until the inotrope is ceased. Patients who have pre-existing congestive cardiac failure or SIRS diagnosed on admission into ICU are at a greater risk of recurrence of AF. Vigilance of magnesium replacement, avoiding excessive volume depletion and the resolution of SIRS would appear a reasonable approach to limit AF recurrence, particularly as many known parameters are managed routinely within a tight range for patients in the ICU. A clear dosing guide is not available and further research is required to elicit the best dosing strategy.

## Abbreviations

AF: Atrial fibrillation
APACHE: Acute Physiology and Chronic Health Evaluation
BMI: Body mass index
DAD: Delayed after-depolarisation
EAD: Early after-depolarisation
ECG: Electrocardiogram
ICU: Intensive care unit
IQR: Interquartile range
SAPS: Simplified Acute Physiologic Score
SIRS: Systemic inflammatory response syndrome
